# *In vitro* assembly of nuclear envelope in tobacco cultured cells

**DOI:** 10.1080/19491034.2021.1930681

**Published:** 2021-05-25

**Authors:** Kentaro Tamura, Haruko Ueda, Ikuko Hara-Nishimura

**Affiliations:** a Department of Environmental and Life Sciences, School of Food and Nutritional Sciences, University of Shizuoka, Shizuoka, Japan; b Faculty of Science and Engineering, Konan University, Kobe, Japan

**Keywords:** BY-2, nuclear envelope, nucleus, endoplasmic reticulum, confocal microscopy

## Abstract

The coordinated regulation of the nucelar envelope (NE) reassembly during cell division is an essential event. However, there is little information on the molecular components involved in NE assembly in plant cells. Here we developed an *in vitro* assay of NE assembly using tobacco BY-2 cultured cells. To start the NE assembly reaction, the demembranated nuclei and the S12 fraction (cytosol and microsomes) were mixed in the presence of GTP and ATP nucleotides. Time–course analysis indicated that tubule structures were extended from the microsomal vesicles that accumulated on the demembranated nuclei, and finally sealed the NE. Immunofluorescence confirmed that the assembled membrane contains a component of nuclear pore complex. The efficiency of the NE assembly is significantly inhibited by GTPγS that suppresses membrane fusion. This *in-vitro assay* system may elucidate the role of specific proteins and provide important insights into the molecular machinery of NE assembly in plant cells.

## Introduction

The nuclear envelope (NE) provides a selective barrier that separates the functional and structural systems of a nucleus from the rest of the cell. It consists of a double membrane, nuclear pore complex (NPC), and nuclear lamina. The outer nuclear membrane forms a continuous membrane system with the endoplasmic reticulum (ER), whereas the inner nuclear membrane (INM) is closely associated with the chromatin and nuclear lamina. During the cell cycle, in plants and animals, the NE typically breaks down at prophase, which allows for open mitosis. At telophase, the NE components reassemble to reestablish the nuclear boundary. The coordinated regulation of the NE reassembly is essential to correct cell functioning [1]. Live cell imaging has demonstrated the dynamic features of plant NE proteins during NE reformation of mitosis in tobacco cultured cells [2,3]. After NE breakdown, INM proteins, SUN1 and SUN2 disperse into the ER membrane and accumulate at the spindle. At telophase, they relocate to the chromatin surface-facing spindle pores and subsequently accumulate at the distal and proximal surface of the chromatin to seal the NE. Other NE proteins, including WIP [4], NMCP [5], and RanGAP [6], were also investigated in terms of their localization during mitosis. However, little is known about the molecular components involved in the NE assembly in plant cells.

*In vitro* NE assembly assays have been well-established using cell-free extracts derived from *Xenopus laevis* eggs [7–11]. The egg extract contains a high concentration of proteins and factors required for *in vitro* nuclei assembly [12]. It leads to extensive membrane fusion events, resulting in NE formation around the sperm chromatin. This assay system has contributed to the independent studies of the individual steps in the assembly mechanism that occur during nuclear formation [12]. For example, it has been demonstrated that an intact tubule ER network is required for NE formation and expansion at the end of mitosis [13,14]. The membrane targeting to chromatin is partially regulated by NE-specific transmembrane proteins that have long basic domains that potentially bind to chromatin [13,15]. The assays have also been widely used to study nuclear transport [16–18], nuclear membrane and NPC assembly [14,19], nuclear breakdown at mitosis [10,20], and many other aspects.

In addition to the *X. laevis* egg system, cell-free NE assembly have been reported in several species, including sea urchins [21], *Drosophila*, [22] and humans [23]. Lu and Zhai developed a heterogeneous NE assembly system using demembranated *X. laevis* sperm and extracts from *Nicotiana tabacum* ovules [24]. However, little is known about the molecular mechanisms underlying plant NE assembly. Since *in vitro* nuclei assembly is comparable to *in vivo* nuclei assembly in fundamental architecture, the cell-free assay is a powerful system to elucidate molecular mechanisms that regulate NE formation. In this study, we have developed a novel assay of plant NE assembly using tobacco BY-2 cultured cells. We also revealed that a membrane fusion inhibitor, GTPγS, suppresses the closing of the NE, suggesting that ER membrane fusion plays an important role in the NE assembly in plants.

## Materials and methods

### Tobacco cultured BY-2 cells

Tobacco (*Nicotiana tabacum*) cultured BY-2 cells were maintained as described previously [25]. Transgenic BY-2 cells, which stably express ER lumen-targeted GFP, were previously generated [25]. The ER lumen-targeted GFP was composed of signal peptide of pumpkin 2S albumin and GFP followed by an ER-retention signal, HDEL.

### Isolation of nuclei from BY-2 cells

Nuclei from BY-2 cells were isolated as described previously with minor modifications [26]. BY-2 cells (5 day old) from 50 ml culture were collected by centrifugation at 1,500 x*g* for 2 minutes and were suspended in 50 ml Cellulase enzyme solution (1% Cellulase ONOZUKA RS, 0.1% pectolyase Y-23, 0.3 M mannitol, 23.4 mM MES-KOH [pH5.7]). The cells were incubated with gentle shaking at 26°C for 4 hours to obtain protoplasts. The protoplasts were filtrated through a 125 µm nylon filter and then centrifuged at 1,000 x*g* for 3 min at 4°C. The pellet of protoplasts was suspended in 15 ml buffer A (0.44 M sucrose, 2.5% ficoll, 5.0% dextran40, 25 mM Tris-HCl [pH8.0], 10 mM MgCl_2_, 3 mM CaCl_2_, 0.01% Triton X-100, 2.5 mM DTT) and homogenized with a glass homogenizer on ice. The homogenate was filtrated through 80 µm nylon filters to remove cell debris. The solution passed through the filters was centrifuged at 1500 x*g* for 10 minutes at 4°C. The pellet was re-suspended in 10 ml 2.3 M sucrose buffer (2.3 M sucrose, 3 mM CaCl_2_, 10 mM MgCl_2_, 25 mM Tris-HCl [pH 8.0], 0.01% TritonX-100) and centrifuged at 65,000 x*g* for 45 minutes at 4°C. The pellet was re-suspended in buffer A and used as “isolated nuclei’.

### An NE formation assay

The isolated nuclei mentioned above were treated with 2% Triton X-100 for 30 min to remove nuclear membrane on ice. The nuclei were washed with buffer B (40 mM PIPES-KOH [pH 7.0], 8 mM EGTA, 1 mM MgCl_2_, 2 mM MnCl_2_, 0.3 M sucrose, 0.5% casein, 2 mM DTT, 2 mM PMSF, 0.2 mg/ml leupeptin) and stained with 5 µM of FM4-64 (Invitrogen) for 10 min at room temperature to confirm whether the nuclei were demembranated. The S12 fraction containing microsomal and cytosolic fraction was isolated according to Yokota et al with minor modifications [27]. Protoplast prepared from 30 ml of BY-2 cells (5 day old) were homogenized in buffer B by passing through a syringe with a 27 G needle. The homogenates were centrifuged at 2,000 x*g* for 4 min at 4°C to remove cellular debris. The supernatants were further centrifuged at 12,000 g for 6 min at 4°C to obtain the S12 fraction. The S12 fraction was premixed with ATP and GTP (final concentrations 3 mM) solution. For inhibition assay, GTPγS (final concentration 3 mM) was added instead of GTP. The demembranated nuclei were added to the mixture to start the NE formation assay on glass slides at room temperature. The nuclei were stained with 1 µg/ml Hoechst 33342. Immunostaining of nuclei with anti-Nup43 antibody [28] was performed as described previously [29]. Fluorescent images were randomly captured at each time point by a confocal laser-scanning microscope (LSM510 META and LSM800) with a 40 × 0.95 N.A. dry objective (Plan-Apochromat, 440654–9902-000, Carl Zeiss) or 20 × 0.8 N.A. dry objective (Plan-Apochromat, 440640–9903-000, Carl Zeiss). We defined ‘nuclei with NE closure’ as a nuclear membrane signal that occupies more than 90% of perimeter of chromatin. In Figure 3(d), we quantified membrane region labeled by GFP of each nucleus (n > 20) and counted nuclei with NE closure. The number of technical replicate is 4 at each time point. Data were processed using ImageJ/Fiji. Three-dimensional (3D) image reconstruction was performed through volume rendering in arivis Vision4D software (Zeiss).

## Results and discussion

### Preparation of demembranated nuclei and cell-free extracts from tobacco BY-2 cultured cells

**Figure 1.**
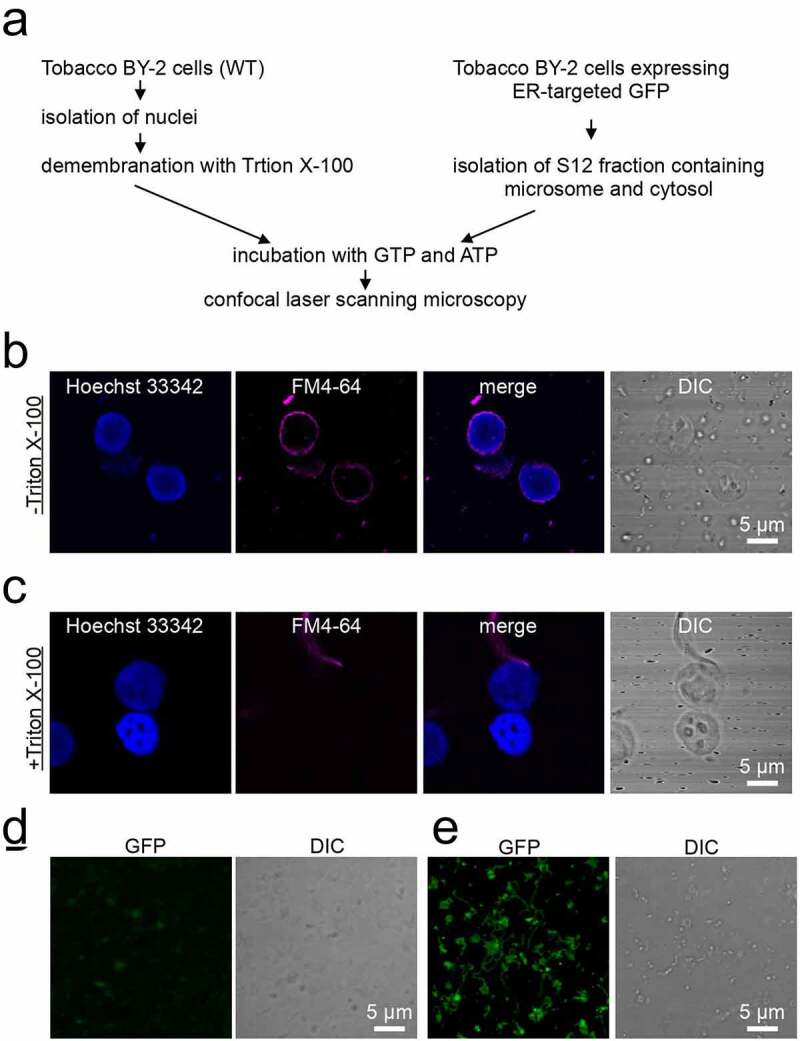
Preparation of demembranated nuclei and S12 fraction from tobacco BY-2 cultured cells. (a) Nuclei were isolated from wild-type tobacco cultured cells and demembranated using Triton X-100. The S12 fraction was isolated from transgenic tobacco cultured cells expressing ER-targeted GFP. The reaction was started by mixing the demembranated nuclei, S12 fraction, GTP, and ATP. The NE assembly was analyzed through confocal microscopy. (b, c) Fluorescence images of isolated nuclei before (b) and after (c) treatment with Triton X-100. Chromatin and nuclear membrane were visualized through Hoechst 33342 and FM4-64 staining, respectively. DIC: differential interference contrast. Bars: 5 µm. (d, e) Fluorescence images of the S12 fraction, which were visualized using ER-targeted GFP, and incubation without (d) and with (e) GTP and ATP. DIC: differential interference contrast. Bars: 5 µm

To establish the *in vitro* NE assembly assay in plants, we isolated nuclei and cell-free extracts (S12 fraction) from tobacco BY-2 cultured cells (Figure 1(a)). Intact nuclei were purified from the cells using a buffer containing a high sucrose concentration [26]. Staining with Hoechst 33342 (a DNA specific dye) and FM4-64 (a membrane specific dye) [30] confirmed that the isolated nuclei have intact nuclear membranes (Figure 1(b)). For the NE assembly assay, nuclei were demembranated with 2% TritonX-100 (a nonionic detergent), resulting in the obliteration of the FM4-64 signal (Figure 1(c)). Demembranation had no effect on nuclear morphology, suggesting that the nuclear matrix remains structurally intact for chromatin organization.

Previously, it has been demonstrated that the S12 fraction of tobacco and *Arabidopsis* cultured cells reconstitutes the ER network *in vitro* [27]. Therefore, we used the S12 fraction as the cell-free extract for *in vitro* NE assembly. The S12 fraction is a subcellular fraction that is rich in microsomes and cytosol, including two classes of myosin XI, which is responsible for ER tubule formation [27]. We prepared the S12 fraction from transgenic tobacco BY-2 cells expressing ER lumen-targeted GFP to visualize NE formation during the assay. During cell fractionation, the fragile ER network was fragmented, resulting in the production of small fluorescent structures with varying sizes and shapes (Figure 1(d)). We confirmed that the S12 fraction was able to form ER tubules and sheets upon ATP and GTP treatment for 30 min on glass slides (Figure 1(e)), which is consistent with previous reports [31]. This result suggests that the S12 fraction from the transgenic tobacco cells is capable of NE reconstitution.

## *An* in vitro*NE assembly assay*

**Figure 2.**
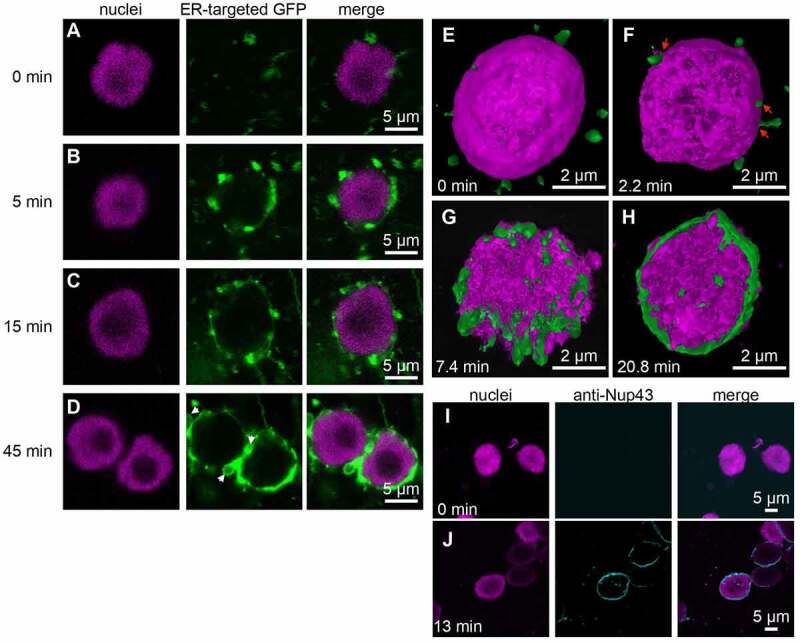
Time–course analysis of NE assembly. (a–d) Representative fluorescence images of the NE assembly assay at each time point. Nuclei were stained with Hoechst 33342 (magenta), and the S12 fraction was stained with ER-targeted GFP (green). White arrows (d) indicate bleb-like structures. Bars, 5 µm. (e–h) 3D volumetric rendering images of NE assembly at each time point. Nuclei were stained with Hoechst 33342 (magenta), and the S12 fraction was stained with ER-targeted GFP (green). Red arrows (f) indicate membranes associated with chromatin. Bars, 2 µm. (i, j) Representative fluorescence images of the NE assembly assay at each time point. Nuclei were stained with Hoechst 33342 (magenta), and the nuclear envelop was stained with anti-Nup43 antibody (cyan). Bars, 5 µm

To start forming the NE, the demembranated nuclei and S12 fraction were mixed with GTP and ATP (Figure 2(a); see Materials and Methods). We observed that small vesicles labeled by ER lumen-targeted GFP were attached to the nuclear periphery after 5 min of incubation (Figure 2(b)). After 15 min, tubule structures were extended from vesicles, and these structures covered the surface of the demembranated nuclei (Figure 2(c)). Membrane closing was completed after 45 min (Figure 2(d)). The excess nuclear membrane formed bleb-like structures (Figure 2(d), arrows). To visualize dynamics of NE formation on the chromatin, the optical sectioning images were assembled into three-dimensional (3D) constructions (Figure 2(e–h)). The 3D volumetric rendering images shows that membrane vesicles and tubules were attached with chromatin surface at an early stage (Figure 2(f), arrows). Subsequently, the membrane was grown to produce sheet-like structures (Figure 2(g)) and nuclear membrane (Figure 2(h)). These results suggest that *in vitro* NE assembly is composed of two steps, namely, vesicle accumulation around the nuclei and subsequent tubule and sheet formation. Immunofluorescence with anti-Nup43 antibody (a marker for NPC) showed that assembled membrane contains NPC (Figure 2(i,j)). Similar results have been obtained by *in vitro* NE assembly using *X. laevis* [32–34] and *Drosophila* [22] extracts. Membrane vesicles were first recruited to the periphery of chromatin in a cytosol-independent manner. The vesicles on the chromatin fused to generate tubules and small cisternae on the chromatin surface [34–36]. This fusion event requires soluble *N*-ethylmaleimide-sensitive factor (NSF) attachment protein receptor proteins and Ran GTPase. The tubules and cisternae subsequently laterally extended to produce larger cisternae and cover the entire chromatin surface, thereby sealing the envelope. Taken together, these results suggest that plant NE formation also requires vesicle fusion on the chromatin to generate tubules.

### An Inhibitory effect of GTPγS on the NE assembly

**Figure 3.**
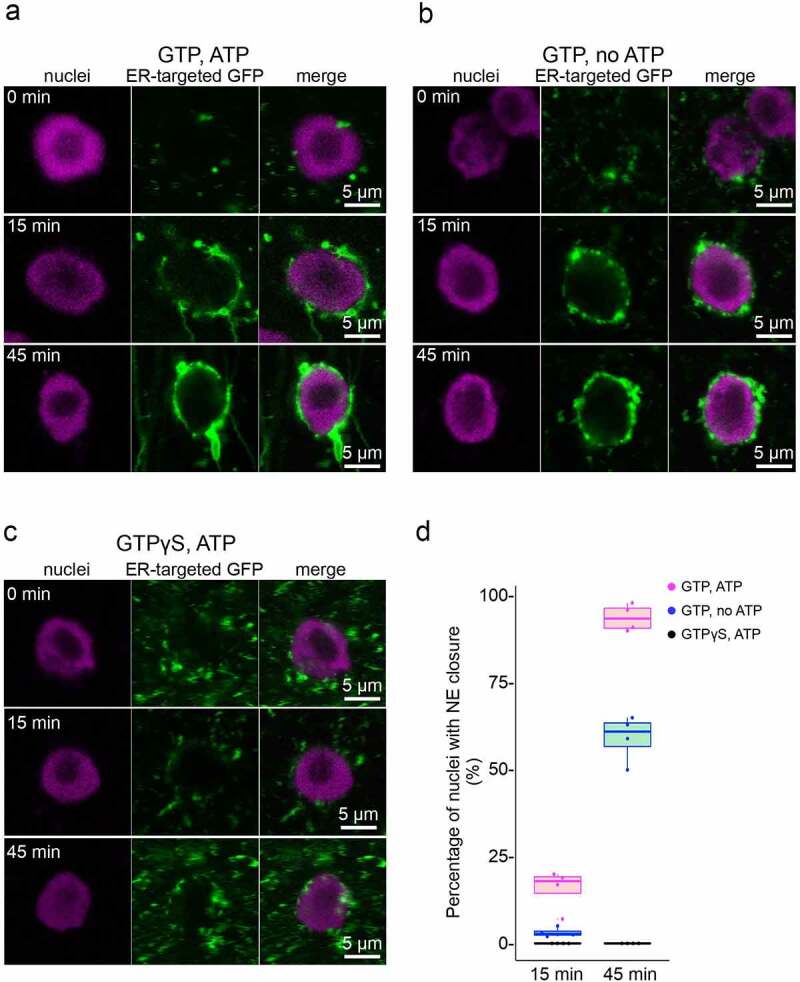
GTP- and ATP-dependent NE assembly. (a–c) Representative fluorescence images of the NE assembly assay in the presence of nucleotides, as indicated in each panel. Nuclei were stained with Hoechst 33342 (magenta), and the S12 fraction was stained with ER-targeted GFP (green). Bars, 5 µm. (d) The percentage of nuclei with NE closure. Each box is bounded by the lower and upper quartiles, the central bar represents the median, the whiskers indicate minimum and maximum values, and beeswarm plots indicate individual data points (technical replicates). The numbers of individual biological experiments are n > 20

We next examined the effects of GTP and ATP on NE assembly in our proposed system. In control experiments containing both GTP and ATP, tubule structures were formed after 15 min, and NE closure (see Materials and methods) occurred in 93.5 ± 3.86% (median ± standard deviation) of nuclei after 45 min of incubation (Figure 3(a,d)). When the reaction was started without ATP, vesicles accumulated on the demembranated nuclei, similar to the control group. However, tubule formation was inhibited after 15 min, compared with the control, leading to a reduction in the closed NE ratio at 45 min (61 ± 6.65%) (Figure 3(b,d)). We also found that a non-hydrolyzable GTP analog (GTPγS) significantly inhibited NE formation (Figure 3(c)). There was no NE formation observed (Figure 3(c,d)), although small vesicles accumulated on the demembranated nuclei surface at 45 min. These results suggest that vesicle accumulation on the demembranated nuclei is independent of GTP and ATP, whereas tubule formation from the vesicles and subsequent efficient NE formation required both GTP and ATP.

The fusion of separated membrane vesicles and fragmented ER require several GTPase proteins in eukaryotic cells [37]. In plant cells, it has been reported that ER tubule formation requires both GTP and ATP and is significantly inhibited by GTPγS *in vitro* [27,31,38]. The activity of GTP hydrolysis was highly induced during ER tubule formation *in vitro* [27]. These results suggest that the inhibition of NE formation by either no ATP or GTPγS was due to a block in reconstituting the tubule network from fragmented ER membranes. Consistent with this finding, it has also been reported that GTPγS and ATPγS efficiently blocked NE formation with a fragmented membrane in an *X. laevis* cell-free system [35], suggesting the evolutionally conserved NE formation system. In tobacco cultured cells, two groups investigated NE reformation during cell division with fluorescent-tagged SUN proteins [2,3]. At telophase, SUN proteins were reassembled at the distal side of nuclei and expanded to the proximal side. Such a polar localization of membrane reformation was not observed in our *in vitro* system. This difference could be caused by the fact that demembranated nuclei were isolated from non-mitotic cells. Further examination will be required to reveal the physiological significance of the polar NE reformation.

In this study, we elucidated the *in vitro* NE assembly system in plants. Unlike previous work [24,39], this system produces ‘pure’ plant nuclei, but not ‘hybrid’ nuclei, that are made from animal (*X. laevis*) and plant (Tobacco and *Orychophragmus*) extracts. The tobacco cultured cells used in this study have a number of advantageous features. First, cells grow fast, making it possible to purify large amounts of cell-free extracts (S12 fraction) and nuclei. Second, several fluorescent marker lines have been generated to visualize membrane dynamics. Third, the cell cycles can be highly synchronized [40], allowing extracts of various cell cycle stages to be prepared. *In vivo* studies of NE assembly in plants are usually limited because NE reconstitution is an essential event for viability during cell cycle. It is expected that *in vitro* NE assembly can generate “biochemically mutant nuclei“ by immunodepletion of specific NE proteins from the reaction to assess the function of each component. The *in vitro* system will provide important insights that help to understand better the molecular machinery of NE assembly in plant cells.
